# Ondansetron induced fatal ventricular tachycardia

**DOI:** 10.4103/0253-7613.43168

**Published:** 2008-08

**Authors:** R. Chandrakala, C.N. Vijayashankara, Kushal K. Kumar, N. Sarala

**Affiliations:** Department of Pediatrics, Sri Devaraj Urs Medical College, Tamaka, Kolar, Karnataka, India; 1 Department of Pharmacology, Sri Devaraj Urs Medical College, Tamaka, Kolar, Karnataka, India

**Keywords:** Ondansetron, ventricular tachycardia

## Abstract

Ondansetron is a serotonin receptor antagonist used widely in the prophylaxis and treatment of postoperative nausea and vomiting (PONV) and vomiting associated with cancer chemotherapy. The common side effects of ondansetron are fever, malaise, diarrhoea, constipation, and allergic reactions. Extra-pyramidal reactions are rare and cardiovascular side effects are even rarer. Even though its clinical safety has been established in a large number of studies, its adverse effects have been reported and these include cardiovascular events like acute myocardial ischemia and arrhythmias in adults.^[1]^  Studies of its adverse effects in children are few. We report a rare adverse effect of ondansetron in a 14-year-old girl, presenting as ventricular tachycardia.

## Case Report

A 14 year olds girl with unremarkable past medical history presented to a local hospital with vomiting and pain in abdomen of eight hours durations.[1] She had received ondansetron 4 mg intramuscularly and an antacid for pain in abdomen. Vomiting persisted and two hours later she developed giddiness. She was referred to Sri Devaraj Urs Medical College Hospital, within three hours from the local hospital. On examination in the emergency room, she was in altered sensorium. She had circulatory failure with cold clammy skin, unrecordable blood pressure and feeble peripheral pulses. Tachycardia was present with heart rate of 180/min.

**Figure 1:**
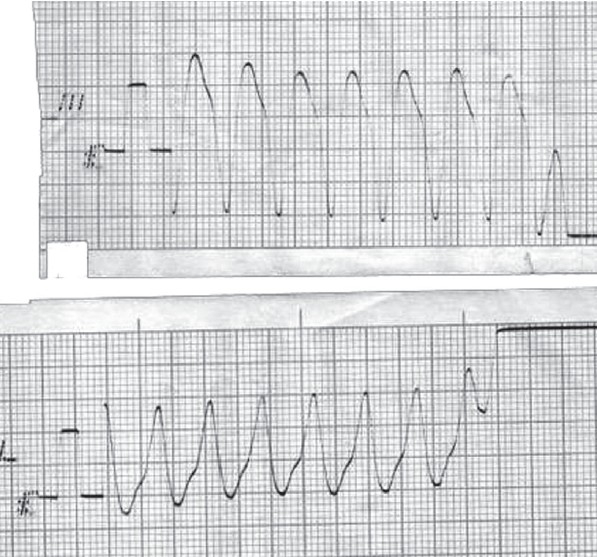
Ventricular tachycardia

Abdominal examination showed no distension and normal bowel sounds. Bilateral crepiataions were observed on auscultation of chest. An ECG showed ventricular tachycardia [Figure 1] and she was treated according to Pulseless Ventricular tachycardia protocol.[2] However, the ventricular tachycardia soon degenerated into ventricular fibrillation resulting in death. Other investigations could not be done as the patient survived only thirty minutes in the hospital. Autopsy was planned, but could not be performed as the parents denied consent.

## Discussion

Ondansetron belongs to a group of 5HT_3_  receptor antagonists used in the management of PONV and cancer chemotherapy induced vomiting.[1] It also decreases the vomiting associated with acute gastroenteritis and the need for hospitalization,[3,4] Its safety and low cost of therapy suggests that it can be valuable in the treatment of gastroenteritis in children.[3] Large studies have established its clinical safety, but some studies in adults have reported adverse effects like myocardial infarction and arrhythmias such as Supra ventricular tachycardia, ventricular tachycardia and atrial fibrillation.[4] Two cases of dysrhythmias have been reported after administration of 4 mg ondansetron.[5] 

Ondansetron is known to cause cardiac arrhythmias by several mechanisms.[1,6] Firstly, voltage dependent Na^+^  and K^+^  channels are determinants of cardiac muscle action potential and the electrocardiogram (ECG). Cardiac Na^+^  channels are responsible for depolarisation and propagation of the cardiac action potential. In ECG, the QRS complex represents the ventricular depolarisation. Two voltage dependent K^+^  channels represented as rapid repolarising current (I_Kr_) and slow repolarising current (I_Ks_) are responsible for rapid and slow repolarisation of cardiac muscle and QT interval in the ECG. Of the two channels, rapid repolarising current is the main repolarising channel in the heart, which is encoded by human ether-a-go-go-related gene (HERG). Ondansetron can block this channel, resulting in lengthening of the repolarisation.[6] Drugs like granisetron and dolasetron act on the Na^+^  and K^+^  channels to prolong QRS or QT interval, resulting in ventricular arrhythmias.[6] The sub-micromolar   affinity of ondansetron for K^+^  channels is possibly responsible for the prolongation of cardiac repolarisation, thus resulting in conduction disturbances.[6] Secondly, the cardiovascular effects of serotonin are mediated by 5HT_1_ , 5HT_2_ , 5HT_3_ , 5HT_4_  receptors, which are distributed throughout the cardio vascular system. 5HT_3_  receptors mediate Bezold-Zarish reflex, which is an autonomic reflex consisting of bradycardia, hypotension and apnea. Suppression of this reflex by ondansetron leads to tachyarrhythmias.[1] Thirdly, in some cases, 5HT_3_  receptor blockade could possibly lead to unopposed action of 5HT_2_  and 5HT_4_  receptors, resulting in tachyarrhythmias and hypertension.[1] 

Though the cause of the death is inconclusive in our case, we presume it was possibly due to ondansetron, because the patient was previously healthy and we could not establish any other cause for ventricular tachycardia. Although she presented with vomiting and pain in the abdomen, she had normal abdomen clinically. She died within fours hours of administration of ondansetron presenting in shock. This cardiogenic shock is secondary to ventricular tachycardia, resulting in ventricular fibrillation. However, the possibility of idiopathic ventricular arrhythmia also cannot be ruled out. Further studies are required to confirm ondansetron induced ventricular arrhythmia and its clinical safety. Till such time, this drug should be used judiciously in patients.
